# Minimal residual disease (MRD) detection in solid tumors using circulating tumor DNA: a systematic review

**DOI:** 10.3389/fgene.2023.1172108

**Published:** 2023-08-10

**Authors:** Lemei Zhu, Ran Xu, Leilei Yang, Wei Shi, Yuan Zhang, Juan Liu, Xi Li, Jun Zhou, Pingping Bing

**Affiliations:** ^1^ Hunan Key Laboratory of the Research and Development of Novel Pharmaceutical Preparations, Changsha, China; ^2^ Academician Workstation, Changsha Medical University, Changsha, China; ^3^ School of Public Health, Changsha Medical University, Changsha, China; ^4^ Geneis Beijing Co., Ltd., Beijing, China; ^5^ Department of Orthopedics, Xiangya Hospital Central South University, Changsha, China

**Keywords:** MRD, tumor, CtDNA, biomarker, NGS

## Abstract

Minimal residual disease (MRD) refers to a very small number of residual tumor cells in the body during or after treatment, representing the persistence of the tumor and the possibility of clinical progress. Circulating tumor DNA (ctDNA) is a DNA fragment actively secreted by tumor cells or released into the circulatory system during the process of apoptosis or necrosis of tumor cells, which emerging as a non-invasive biomarker to dynamically monitor the therapeutic effect and prediction of recurrence. The feasibility of ctDNA as MRD detection and the revolution in ctDNA-based liquid biopsies provides a potential method for cancer monitoring. In this review, we summarized the main methods of ctDNA detection (PCR-based Sequencing and Next-Generation Sequencing) and their advantages and disadvantages. Additionally, we reviewed the significance of ctDNA analysis to guide the adjuvant therapy and predict the relapse of lung, breast and colon cancer et al. Finally, there are still many challenges of MRD detection, such as lack of standardization, false-negatives or false-positives results make misleading, and the requirement of validation using large independent cohorts to improve clinical outcomes.

## 1 Introduction

Liquid biopsy, which has many advantages such as non-invasiveness, acceptability, repeatability and prediction of tumor burden and treatment response, has played an increasingly important role in the diagnosis and treatment of cancer. Cancer biomarkers can be extracted and analyzed from the blood, urine, pleural effusion, seroperitoneum, cerebrospinal fluid or saliva of cancer patients with this novel detection method. Circulating tumor cells (CTCs), cell free nucleic acids, exosomes and other biological components secreted into body fluids by cancer cells are all analytes of liquid biopsies, providing biomarkers such as somatic point mutations, amplifications, deletions, gene fusions, DNA methylation markers, miRNAs, proteins, and metabolites.

Cell-free DNA (cfDNA) consists of double-stranded DNA with a length of 150–200 base pairs that circulate mainly in the blood, released through apoptosis, necrosis, and phagocytosis (Corcoran and Chabner, 2018). The origin of cfDNA is hemopoietic cells such as erythrocytes, leukocytes and endothelial cells in healthy individuals, and normal tissues damaged by ischemia, trauma, infection or inflammation can also contribute cfDNA (Schwarzenbach et al., 2011; Snyder et al., 2016). Circulating tumor DNA (ctDNA) is a rather minor fraction of cfDNA released by malignant tumors into the bloodstream or other bodily fluids (Diehl et al., 2008). ctDNA is shorter compared to cfDNA derived from non-cancer cells (Mouliere et al., 2011; Zheng et al., 2012; Underhill et al., 2016). ctDNA is generally more fragmented than non-mutant cfDNA, with a maximum enrichment between 90 and 150 bp compared with 250–320 bp (Bao et al., 2016; Mouliere et al., 2018; He et al., 2020a). ctDNA levels correlate with clinical and pathological features of cancer, including stage, tumor burden, localization, vascularization, and response to therapy (Diehl et al., 2008; Bettegowda et al., 2014; Heitzer et al., 2019). In addition, ctDNA levels vary according to tumor type, shedding rate, and other biological factors (Bettegowda et al., 2014; Siravegna et al., 2019).

**FIGURE 1:**
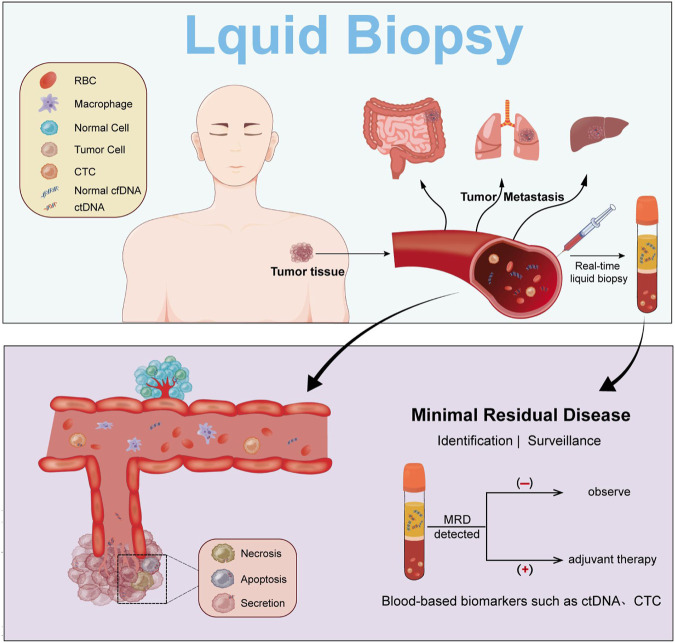
ctDNA during cancer progression. Detection of ctDNA is achieved by liquid biopsy, which allows monitoring and adjunctive treatment of MRD.

MRD (Minimal residual disease) is defined as a small number of cancer cells that remain in the body after cancer treatment (those that do not respond to treatment or are resistant to drugs), which may ultimately lead to disease relapse. ctDNA tests can benefit patients with solid tumor for its capacity to confirm the existence of MRD during the postoperative period. On the other hand, MRD tests can monitor and assess the biomarkers that indicate the effectiveness of adjuvant chemotherapy as well as drug resistance (Guibert et al., 2020). (Figure 1)

## 2 ctDNA detection methods

The ctDNA detection methods are mainly divided into two main categories: the PCR-based detection methods such as Droplet Digital PCR (ddPCR), and the Next-Generation Sequenceing (NGS). These methods have significant differences in detection sensitivity, specificity and coverage.

### 2.1 Droplet digital PCR

Droplet Digital PCR (ddPCR) is to distribute the DNA sample into millions of water-oil emulsion droplets before the traditional PCR amplification, which means, each of the droplets either contains no gene under test or contains one gene. After PCR amplification, each microdroplet was detected one by one. The initial copy number or concentration of the DNA to be tested could be obtained according to the Poisson distribution principle and the number and proportion of positive microdroplet (Zonta et al., 2016; He et al., 2021). The ddPCR provides an absolute quantification that improves sensitivity at a low cost, and it can also achieve high specificity by designing the primers and probes individually.

The detection limit of ddPCR turns out to be about 0.1% (Dong et al., 2018; Corless et al., 2019; He et al., 2020b; Vessies et al., 2020). Reported LODs vary due to differences in the amount of ctDNA in plasma, sample quality, and analysis approaches. Although ddPCR is very effective for detecting small numbers of mutations identified from sequencing of tumor tissue or hot-spot mutations with a high prevalence, this detection approach has inferior clinical sensitivity for MRD than highly parallel NGS methods monitoring multiple mutations (Garcia-Murillas et al., 2015; Pietrasz et al., 2017; Schøler et al., 2017; Christensen et al., 2018).

The ddPCR method has the advantages of low cost and short detection time, but the disadvantage of detecting only known variants and analyzing only a limited number of variants localize its use in clinical practice as a supplement or conditional substitute for tissue biopsy for genotyping (Elazezy and Joosse, 2018; Wei et al., 2018; Franczak et al., 2019; Kerachian et al., 2019; Peng et al., 2022; Wang et al., 2022). Therefore, in most circumstances, ddPCR is not the preferred approach for solid tumor MRD detection.

### 2.2 PCR amplicon-based NGS

Next-generation sequencing (NGS) is a high-throughput technique that enables detection of billions of DNA molecules from a biological sample. Compared with ddPCR method, NGS can search for previously unidentified variations (Wan et al., 2017). With the emergence of more and more therapeutic related molecular targets, NGS has become increasingly important in cancer research. Although whole genome sequencing (WGS) or whole exome sequencing (WES) can provide more detailed genomic information, ctDNA NGS techniques for clinical application utilize amplicon-based NGS or hybridization capture-based NGS to provide clinically relevant information with higher sequencing depth at lower cost.

Amplicon-based NGS is one of the popular detection methods to identify specific ctDNA molecules. Gene-specific PCR amplicons are used to amplify the specific genomic regions originated from tumor-derived mutations before NGS is performed. Unique molecular identifiers (UMI) can help increasing sensitivity and specificity of NGS detection (Phallen et al., 2017; Goldberg et al., 2018). Forshew et al. first described Tagged-Amplicon Sequencing (TAm-Seq), demonstrating that cancer mutations with allele frequencies as low as 2% and sensitivity and specificity over 97% could be detected, and this technique was successfully applied to cancer mutation surveillance in patients with advanced ovarian cancer (Forshew et al., 2012). Later on, TAm-seq was used to apply NGS to a target panel which has detection limits as low as 0.01% (Gale et al., 2018). Although amplicon-based targeted NGS methods are highly sensitive and specific, amplification may potentially bias the observed mutant allele, and this technique is limited to the queried amplicon space while mutation detection performed (Chaudhuri et al., 2015; Wan et al., 2017; Abbosh et al., 2018).

### 2.3 Hybridization capture-based NGS

Hybrid capture-based NGS, which hybridize relevant DNA sequences to biotinylated probes before NGS is performed, is developed to improve the detection of multiple mutations of tumor with high sensitivity and without significant prior knowledge (Wan et al., 2017).

Newman et al. utilized an highly sensitive ctDNA detection technique named Cancer Personalized Profiling by Deep Sequencing (CAPP-Seq) (Newman et al., 2014a). Cancer WGS and WES data from databases such as The Cancer Genome Atlas (TCGA) and the Catalog of Somatic Mutations in Cancer (COSMIC) was used for bioinformatics analysis to identify recurrently mutated genomic regions in the population of a given cancer type. Biotinylated probes, designed according to these results, were applied to cfDNA capture before NGS of certain cancer patients in order to quantitate the ctDNA with a detection limit of 0.02%. UMI was used to reduce the effect of PCR errors and a bioinformatic error correction step called polishing was used to reduce the effect of stereotypical background artifacts (Newman et al., 2016). Recently ctDNA detection limit was improved to 1 part per million by utilizing multiple somatic mutations within individual DNA fragments to reduce the effect of both technical and biological errors (Kurtz et al., 2021).

The capability of CAPP-Seq includes detection of SNV, insertions/deletion (indel), and genomic rearrangements without individuation. Compared to amplicon-based NGS, Capp-seq shows more reliable detection of copy number changes and allows detection of fusion proteins (Gagan and Van Allen, 2015; Xiao et al., 2022). Otherwise, results from sequencing can reveal the mechanisms of carcinogenesis and drug resistance (Chabon et al., 2016; Khan et al., 2018).

### 2.4 Whole genome sequencing (WGS) and whole exome sequencing (WES)

NGS approaches have become prevalent for tumor sequencing and have also been applied to ctDNA detection. WGS applied to cfDNA achieves a sequencing depth of 0.1× and WES achieves a sequencing depth of 100× (Farris and Trimarchi, 2013; Heitzer et al., 2013; Murtaza et al., 2013; Cohen et al., 2017). Although some studies suggest that WGS is feasible for clinical application to certain patients, it is prohibitive for routine clinical implementation of WGS because of its cost and time required to perform WGS and the associated bioinformatic analysis (Welch et al., 2011; Chan et al., 2013). Therefore, WES turns out to be feasible to improve detection sensitivity and reduce cost while maintaining comprehensive coverage of likely mutated genomic regions. The exons are enriched for most of the pathogenic somatic mutations while they represent only 1.5% of the whole genome (Choi et al., 2009). Above all, there is an inverse correlation between sequencing breadth and detection cost, and sequencing depth *versus* detection limit of detection. Due to the low level of ctDNA in body fluids, targeted approaches, including hybridization capture-based NGS, PCR amplicon-based NGS, are superior to more extensive sequencing approaches such as whole exome or whole genome sequencing.

## 3 Application of ctDNA for MRD detection

ctDNA detection has shown promising clinical potential as a method to detect MRD in solid tumors after radical therapy and before clinical or radiographic disease recurrence (Chaudhuri et al., 2017). MRD status is closely associated with future radiological relapse and the detection of ctDNA after clinical treatment may improve the decision of the next therapeutic regimen. Most treatments are still based on strict chemotherapy regimens, although the probability of serious adverse effects is lower than in the past. Therefore, it is important to avoid unnecessary adjuvant chemotherapy when it can be established that patients may not benefit. ctDNA analysis showed that MRD was associated with poor prognosis in patients with malignant tumors. In this review, we focus on the significance of ctDNA analysis to guide the adjuvant therapy and predict the relapse of lung, breast and colon cancer.

### 3.1 Lung cancer

Among patients with non-metastatic lung cancer, some patients can be cured by primary surgical resection, radiotherapy and comprehensive treatment including chemotherapy (Kalemkerian et al., 2013; Ettinger et al., 2022). In fact, by the time recurrent or progressive lesions were detected by imaging tests after treatment, the patient’s systemic tumor burden was significantly increased. Therefore, there is a great interest in whether MRD detection after radical resection of NSCLC can identify patients at risk of recurrence and provide personalized adjuvant therapy before the tumor burden increases (Kalemkerian et al., 2013; Chaudhuri et al., 2017; Chen et al., 2017; Chen et al., 2019a; Zhao et al., 2019; Peng et al., 2020; Ettinger et al., 2022).

The TRACERx study showed that MRD was predictive of recurrence before routine imaging and that more than 99% of MRD-negative patients did not relapse after treatment (Abbosh et al., 2017). The time interval between the increase in ctDNA levels after surgery and the clinical diagnosis of cancer recurrence provides an opportunity for clinical intervention.

Dynamic study prospectively revealed the dynamic changes of ctDNA in patients with primary lung cancer after surgery (Chen et al., 2019b). After tumor resection, ctDNA level decreased rapidly in patients with surgical lung cancer. The half-life of ctDNA after radical resection of lung cancer is only 35 min. They proposed that 3 days after R0 resection can be used as a baseline for postoperative monitoring of lung cancer.

Chaudhuri et al. (Chaudhuri et al., 2017) introduced their research utilizing CAPP-seq to detect ctDNA. After 36 months of MRD detection, 100% of the patients with detectable ctDNA had disease progression, while 93% of those without detectable ctDNA had no progression of cancer (HR = 43.4, *p* < 0.001). The long-term survival rate of patients without ctDNA detected in MRD was significantly higher than that of patients with ctDNA detected (*p* < 0.001). They suggested that both node-positive and node-negative patients with stage I to III NSCLC may benefit from personalized adjuvant therapy. Patients without tissue material may benefit from tyrosine kinase inhibitors (TKI) or immune checkpoint inhibitors (ICI) with assessing actiable mutations and mutational burden in ctDNA.

In the study of Kuang’s, they detected tumor tissue-specific mutated ctDNA in preoperative plasma samples from 19 (50%) patients (Kuang et al., 2020), and preoperative ctDNA in plasma was consistent with that in tissue. Compared with patients with undetectable ctDNA after chemotherapy, the RFS of ctDNA-positive patients after chemotherapy was worse (HR = 8.68, *p* = 0.022). ctDNA-negative patients after chemotherapy had better long-term efficacy than patients with positive ctDNA after chemotherapy (HR = 4.76, *p* = 0.047).

Gale et al. reported their study using patient-specific assays with up to 48 amplicons targeting tumour-specific variants unique to each patient to monitor postoperative MRD (Gale et al., 2022). Of the 48 patients whose samples were collected 1–3 days after surgery, ctDNA was detected in 12 samples (25%), with a median eVAF of 0.0026%. Therefore, in the case of complete excision of the disease, ctDNA may be present transiently in the blood at low concentrations. ctDNA was detectable in 18/28 (64.3%) patients with clinical recurrence of primary tumors. ctDNA detection had clinical specificity >98.5% and preceded clinical detection of relapse of the primary tumour by a median of 212.5 days. They suggested that MRD detection may be best delayed beyond the first few days as well, because ctDNA was detectable during 1–3 days after surgery in 25% patients, but half of them did not have clinical relapse.

A recent study identified a potentially cured population of localized NSCLC by longitudinal MRD detection (Zhang et al., 2022). From 261 patients with stage I to III NSCLC who underwent definitive surgery, 913 peripheral blood samples were successfully detected by MRD assay. In the surveillance population, only 6 patients (3.2%) with longitudinally undetectable MRD relapsed, with a negative predictive value of 96.8%. The authors identified these patients with longitudinally undetectable MRD as potentially cured patients. The peak risk for detectable MRD was approximately 18 months after the landmark detected. The positive predictive value of longitudinal detectable MRD was 89.1%, and the median lead time was 3.4 months. MRD detection is not ideal for the monitoring of patients with only brain recurrence (n = 1/5, 20%). Further subgroup analysis showed that patients with undetectable MRD may not benefit from adjuvant therapy. In addition, the authors suggest that the risk of developing detectable MRD decreased progressively 18 months after the biomarker discovery.

In conclusion, MRD detection can identify patients at risk of recurrence earlier and is a practical prognostic factor after radical NSCLC surgery. Positive ctDNA after treatment may indicate the presence of MRD, which may be a signal suggesting a change in treatment regimen. After treatment, ctDNA can change from positive to negative, which means that surgery or adjuvant therapy can remove MRD, thereby changing disease progression and survival.

### 3.2 Breast cancer

Although tumor biopsy has long been the standard method for tumor detection, its limitations have made minimally invasive and relatively inexpensive liquid biopsy an alternative. For patients with early-stage breast cancer, ctDNA testing can monitor tumor burden and treatment response, so as to guide therapeutic regimen selection.

Riva et al. described their study that massively parallel sequencing (MPS) was performed on patients with nonmetastatic triple-negative breast cancer (TNBC) and droplet digital PCR (ddPCR) was used to monitor TP53 mutations expressed in tumor tissues (Riva et al., 2017). Patients were treated with neoadjuvant chemotherapy prior to surgery, ctDNA levels decreased rapidly during NCT and no MRD was detected postoperatively. The slow decline in ctDNA levels during NCT is closely associated with shorter survival.

Another study used whole exome sequencing to detect mutations in tumor tissue (Parsons et al., 2020). They then performed an individualized MRD assay to detect mutations in cfDNA. This approach was 100-fold more sensitive than ddPCR when tracking individual mutation. MRD detection at 1 year was strongly associated with distant recurrence (HR = 20.8; 95% confidence interval, 7.3–58.9). The median lead time from first detectable ctDNA to clinical recurrence was 18.9 months.

In the neoadjuvant I-SPY 2 TRIAL, cfDNA was isolated from 291 plasma samples of 84 high-risk early breast cancer patients (Magbanua et al., 2021). 16 patient-specific mutations were identified by whole exome sequencing of pretreated tumors, and then ultra-deep sequencing of cfDNA from patients was performed with this personalized ctDNA detection panel. Patients with positive ctDNA after 3 weeks of neoadjuvant chemotherapy had a significantly lower probability of pathological complete response (pCR) after treatment than patients with negative ctDNA (odds ratio 4.33, *p* = 0.012). All patients who achieved pCR were ctDNA negative after neoadjuvant chemotherapy (n = 17, 100%). While ctDNA-positive patients (14%) who failed to achieve pCR (n = 43) showed a significantly high risk of metastatic relapse [HR 10.4; 95% CI 2.3–46.6]. 86% of those who did not achieve pCR and had negative ctDNA had a favorable prognosis. The author suggested that even in patients who did not achieve pCR, insufficient ctDNA clearance was an important predictor of poor treatment response and metastatic tumor recurrence, and clearance was associated with improved survival.

In advanced or metastatic tumors, ctDNA has high clinical value and development prospects because of its relatively high detection rate (Diehl et al., 2008; Tie et al., 2015; Jiang et al., 2022). Recently, Liu et al. introduced their research of metastatic breast cancer (Liu et al., 2022a). They established a novel ctDNA-level Response Evaluation Criterion in Solid Tumors (ctle-RECIST) to assess treatment response and predict progression-free survival (PFS) based on ctDNA alteration levels and variant allele frequency (VAF). By monitoring and analyzing the ctDNA of 223 patients with metastatic breast cancer at different time points before and after treatment, the results showed that the median PFS of patients without ctDNA changes was significantly longer than that of patients with ctDNA changes (6.63 vs 4.9–5.7 months, *p* < 0.05). In addition, they found that ctDNA detection may be a good complement to radiological assessment, due to the median PFS of double DCR group tended to be longer than that of single DCR group [HR 1.41 (0.93–2.13), *p* = 0.107].

In the treatment of breast cancer patients, PARP inhibitors are synthetically lethal to TNBC tumors carrying BRCA1/2 aberrations by impairing DNA repair mechanisms (Helleday et al., 2005). Genomic alterations detected by longitudinal plasma sampling can identify genes that are resistant to PARP inhibitors such as olaparib and velipariib. Mutations in the TP53 and PIK3CA gene in ctDNA have been sensitive and specific circulating blood biomarkers (Dawson et al., 2013). In addition, ESR1-mutated ctDNA has also been identified as a predictive marker of response to aromatase inhibitor therapy (Guttery et al., 2015; Schiavon et al., 2015). These studies suggest that ctDNA detection can be used to track molecular alterations in patients before and after treatment to develop personalized targeted therapies.

### 3.3 Colorectal cancer

Compared with the lack of sufficient tumor tissue in the specimen and the need for a long test cycle in the tissue biopsy, the utilization of liquid biopsy to detect ctDNA is expected to become an effective tool to promote precision medicine.

For stage II CRC patients, most of them did not receive postoperative chemotherapy. MRD detection is needed to identify 10%–15% of those patients who still have residual lesions after surgery (Osterman and Glimelius, 2018). Postoperative chemotherapy may help reducing the risk of relapse for those who have positive ctDNA. For stage III CRC patients, 30% of them had clinical recurrence after receiving postoperative chemotherapy (Osterman and Glimelius, 2018). At the same time, most patients with stage III colorectal cancer receive postoperative chemotherapy, although more than 50% of patients are cured by surgery (Böckelman et al., 2015; Påhlman et al., 2016; Babaei et al., 2018). Therefore, MRD detection is one potential approach to address the problem of how to better identify patients who could benefit from postoperative adjuvant therapy.

In a previous study, 40% of patients with stage II colorectal cancer who received 6 months of conventional adjuvant chemotherapy had an absolute risk reduction of only 3%–5%, despite the risk associated with potentially serious adverse events and without means to monitor the efficacy of adjuvant therapy (Wirtzfeld et al., 2009). In another study of patients with stage III colorectal cancer, at least one somatic mutation was identified in tumor tissue from all 96 evaluable patients (Tie et al., 2019). ctDNA was detectable in 20 of 96 (21%) postoperative samples and was associated with poor recurrence-free survival (HR, 3.8; 95% CI, 2.4–21.0; *p* < 0.001). For patients received chemotherapy, 15 of 88 (17%) samples were ctDNA positive, with a 30% estimated 3-year RFI. While for those ctDNA undetectable, the 3-year RFI was 77% (HR, 6.8; 95% CI, 11.0–157.0; *p* < 0.001). The author found out that postoperative ctDNA status was independently associated with RFI and significantly outperformed standard clinicopathologic characteristics as a prognostic marker. They later utilized meta-analysis to summarize their previous studies and concluded that the 5-year recurrence-free rate and overall survival rate of patients with non-metastatic CRC who had detectable ctDNA after surgery were poorer (Tie et al., 2016; Tie et al., 2019; Tie et al., 2019; Tie et al., 2021). In this meta-analysis, they combined individual patient data from three independent cohort studies of non-metastatic colorectal cancer (CRC). A massively parallel sequencing platform SafeSeqS was used to analyze ctDNA from 485 CRC patients. ctDNA was detected in 59 (12%) patients postoperatively and the risk of recurrence increases exponentially with increasing ctDNA mutation allele frequency (MAF) (HR, 1.2, 2.5 and 5.8 for MAF of 0.1%, 0.5% and 1%). ctDNA was detected in 3 of 20 patients (15%) with local regional recurrence and 27 of 60 patients (45%) with distant recurrence (*p* = 0.018). This also implies that ctDNA is a better predictor of distant recurrence than local regional recurrence.

An observational GALAXY study recently analyzed MRD in patients with stage I-Ⅳ colorectal cancer (Taniguchi et al., 2021; Kotaka et al., 2022). Within the 188 MRD-positive patients, 95 received postoperative adjuvant chemotherapy. ctDNA levels decreased at a significantly faster rate in patients who received adjuvant chemotherapy than in those who did not receive adjuvant chemotherapy (68% vs. 7%; HR: 17.1; *p* < 0.001). Furthermore, patients received adjuvant chemotherapy had significantly longer 6-month DFS than those who did not (84% vs. 34%; HR: 0.15; *p* < 0.001).

In a recent study, Liu et al. used a technique that allows multiple tests of one single cfDNA sample using different methods (Liu et al., 2022b). They detected MRD using 3 approaches for each sample: personalized detection targeting tumour-informed mutations, universal panel for genes frequently mutated in colorectal cancer (CRC), and low depth sequencing for copy number alterations (CNAs). MRD positivity on personalized detection after neoadjuvant therapy was significantly associated with an increased risk of recurrence (HR = 27.38; *p* < 0.0001). Post-nat universal Panel was good at predicting recurrence in patients with high clinical risk, but not in patients with low clinical risk. CNAs analysis also showed a compromised performance in predicting recurrence.

Current methods for monitoring disease status in patients with metastatic colorectal cancer include radiographic imaging techniques and detection of serum CEA levels. However, serum CEA levels may be increased in only 70%–80% of patients (Goldstein and Mitchell, 2005).

In a study of patients with metastatic colorectal cancer (Garlan et al., 2017), ≥80% ctDNA clearance after first-line or second-line chemotherapy was associated with significantly improved objective response rates (47.1% vs. 0%; *p* = 0.003) and longer median PFS (8.5 months vs. 2.4 months; HR 0.19, 95% CI 0.09–0.40; *p* < 0.0001) and OS (27.1 months vs. 11.2 months; HR 0.25, 95% CI 0.11–0.57; *p* < 0.001). In another study, the authors used amplicon based deep sequencing to detect ctDNA in mCRC patients (Osumi et al., 2019). Patients with lower ctDNA levels (≤50%) showed significantly longer PFS and OS than patients with higher ctDNA levels (>50%) 8 weeks after initiation of chemotherapy.

In patients with stage II and III CRC, based on current studies, it has been demonstrated that ctDNA may be a useful prognostic marker after surgery to guide initial adjuvant therapy and monitor postoperative recurrence. ctDNA analysis can potentially transform the postoperative management of CRC by enabling risk stratification, chemotherapy monitoring, and early recurrence detection.

### 3.4 Other tumors

In recent studies, ctDNA has emerged as a potential biomarker for minimal residual disease (MRD) after treatment of many solid tumors (Lou et al., 2018; Xiong et al., 2019; He et al., 2020c; Chen et al., 2020; Xu et al., 2020). In a study of patients with locally advanced unresectable or metastatic gastric cancer, patients with low ctDNA levels significantly prolonged DFS after the first cycle of chemotherapy (3 months) compared with patients with high ctDNA levels (COX regression *p* = 0.0228) (Normando et al., 2018). In another study, advanced gastric cancer patients with higher ctDNA levels were more likely to have peritoneal recurrence and significantly lower 5-year overall survival rate than patients with lower ctDNA levels (39.2% vs 45.8%, *p* = 0.039) (Fang et al., 2016). Carrying ctDNA mutations was associated with poor prognosis among patients with late stage gastric cancer. In a study of gastrointestinal malignancies, ctDNA levels were higher in the gastrointestinal tumor group than in the carcinoma *in situ* group and healthy controls (*p* = 0.019) (Lan et al., 2017). For recurrent gastric cancer, persistent high levels of ctDNA and an increasing trend were observed after surgery (Wen et al., 2015). In addition, ctDNA levels tended to be more sensitive than CEA levels in predicting recurrence during postoperative monitoring.

In a study of metastatic gastroesophageal cancer, ctDNA was detectable in plasma before treatment in 75% of 72 patients and correlated well with mutations on metastatic biopsy (86% agreement) (van Velzen et al., 2022). The detection of multiple mutations in baseline plasma ctDNA was associated with poorer overall survival (OS, HR 2.16, 95% CI 1.10–4.28; *p* = 0.027) and PFS (PFS, HR 2.71, 95% CI 1.28–5.73; *p* = 0.009), and the VAF was associated with baseline tumor volume (Pearson’s R 0.53, *p* < 0.0001). In addition, patients with residual ctDNA detected after 9 weeks of treatment had worse OS and PFS (OS: HR 4.95, 95% CI 1.53–16.04; *p* = 0.008; PFS: HR 4.08, 95% CI 1.31–12.75; *p* = 0.016).

A large proportion of the patients with early and intermediate stage liver cancer after surgery will have recurrence. In a recent study, peripheral blood samples were collected from all patients after surgery and analyzed by next-generation sequencing based on hybrid capture (Ye et al., 2022). The recurrence rates of ctDNA positive group and ctDNA negative group were 60.9% and 27.8%, respectively. Multivariate Cox regression analysis showed that postoperative ctDNA was an independent prognostic factor for DFS (HR: 6.074, 95% Cl: 2.648–13.929, *p* < 0.001) and OS (HR: 4.829, 95% CI: 1.508–15.466, *p* = 0.008). The prognosis of patients with negative ctDNA was better than that of patients with positive ctDNA regardless of tumor stage. In addition, the authors suggested that the combination of ctDNA and AFP detection could improve the prediction performance.

The value of ctDNA in predicting early postoperative tumor recurrence and monitoring tumor burden in patients with hepatocellular carcinoma (HCC) was investigated in another prospective study (Zhu et al., 2022). They utilized NGS to analyze the ctDNA sequences before and after surgery, and whole exome sequencing was used to detect the DNA of HCC and adjacent tissues. During a median follow-up of 17.7 months, 9 patients (22%) experienced cancer relapse. The positive rate of ctDNA in the non-recurrence group was significantly lower than that in the recurrence group, and ctDNA positivity was associated with significantly shorter recurrence-free survival (RFS). The author suggested that median VAF of baseline ctDNA was an independent predictor of RFS in HCC patients.

Pancreatic cancer is an aggressive solid tumor with a poor prognosis. Currently used biomarkers that are often used to identify advanced pancreatic cancer also do not indicate prognosis. A recent study used hybrid capture-based NGS to sequence ctDNA in patients with metastatic pancreatic cancer (Guan et al., 2022). In 40 tumor tissue samples, mutations in KRAS (87.5%, N = 35) and TP53 (77.5%, N = 31) were more common, and ≥3 mutations in driver genes were strongly associated with overall survival (OS). Univariate analysis showed a significant association between CDKN2A or SMAD4 mutation in ctDNA and PFS in 35 blood samples. Cox hazard proportion model showed that CDKN2A mutation in ctDNA (HR = 16.1, 95% CI = 4.4–59.1, *p* < 0.001) were significantly associated with OS. Patients’ CDKN2A mutation in ctDNA (HR = 6.8, 95% CI = 2.3–19.9, *p* = 0.001) and SMAD4 mutation (HR = 3.0, 95% CI = 1.1–7.9, *p* = 0.031) were significantly associated with PFS. Disease progression detected by ctDNA was 0.9 months earlier than radiological imaging (mean PFS: 4.6 m vs. 5.5m, *p* = 0.004).

In another research of patients with borderline resectable pancreatic cancer, no significant decrease in median RFS or OS was observed in ctDNA-positive patients before treatment or after NAC (Kitahata et al., 2022). The median OS of patients (723 days) with positive ctDNA was significantly shorter than that of patients with negative ctDNA (not reached; *p* = 0.0148). The hazard ratio for adjusted survival risk increased from 4.13 times to 17.71 times for patients with a risk factor (detectable ctDNA or CA19-9>37 U/ml) compared with patients without risk factors (both *p* = 0.0055).

Perioperative systemic chemotherapy can improve the prognosis of upper tract urothelial carcinoma (UTUC). A recent study utilized NGS to analyze perioperative ctDNA to identify patients with poor prognosis who require perioperative chemotherapy (Nakano et al., 2022). They performed targeted ultra-deep sequencing of plasma free DNA (cfDNA) and albugemma DNA, as well as whole-exome sequencing of cancer tissue, thereby eliminating possible false positives. ctDNA was positive in 23 of 50 untreated UTUC patients (46%) and in 17 of 43 localized UTUC patients (40%). Among preoperative risk factors, only preoperative ctDNA score >2% was a significant and independent risk factor associated with poor recurrence-free survival (RFS). In addition, the presence of ctDNA early after surgery was significantly associated with poor RFS, suggesting the presence of MRD.

In another study of urothelial carcinoma, the authors improved the performance of the prognostic model by combining ctDNA sequence aggregate VAF (aVAF) values with clinical factors, including age, sex, and liver metastases (KyrillusShohdy et al., 2022). In consecutive ctDNA samples, an increase in ctDNA aVAF of ≥1 predicted disease progression within 6 months in 90% of patients. The majority of patients with aVAFs≤0.7 in three consecutive ctDNA samples achieved a durable clinical response (≥6 months).

## 4 Challenges of MRD detection

When we perform MRD detection, the number of specific variants we focused on was very small because the total number of gene copies in the plasma samples was limited. As we all know, MRD detection often requires a high sequencing depth, the sensitivity of ctDNA analysis is limited, and when VAF lowers close to LOD, the number of specific variants in the sample may be demanding. In addition, the tumor fraction of cfDNA varies between cancer entities and even between patients with the same cancer entity (Bachet et al., 2018; Normanno et al., 2018; Jiang and Yan, 2021; Huo et al., 2022). In some ctDNA-based studies, it has been found that tumor micrometastases represent a higher tumor burden than residual local disease, and therefore can shed higher ctDNA levels (Azad et al., 2020; Tie et al., 2021). Therefore, some false-negative results cannot be prevented due to biological factors such as low DNA shedding in some tumors or the location of the metastasis itself. The sensitivity of different types of mutations is also different. The ability of different techniques to detect single nucleotide mutations differs from that of structural variants (e.g., fusion) or copy number variants (e.g., copy number amplification). ctDNA analysis is less sensitive to detect structural variants or copy number variants. Therefore, the interpretation of ctDNA results needs to take into account that the amount of ctDNA may not be sufficient to detect specific types of variation. Pascual J, Attard G, Bidard FC, Curigliano G, De Mattos-Arruda L, Diehn M, Italiano A, Lindberg J, Merker JD, Montagut C, Normanno N, Pantel K, Pentheroudakis G, Popat S, Reis-Filho JS, Tie J, Seoane J, Tarazona N, Yoshino T, Turner NC. ESMO recommendations on the use of circulating tumour DNA assays for patients with cancer: a report from the ESMO Precision Medicine Working Group. Ann Oncol. 2022 August; 33(8):750–768. doi: 10.1016/j.annonc.2022.05.520. Epub 2022 July 6. PMID: 35809752.

DNA fragments from the clonal hematopoiesis of indeterminate potential (CHIP) or non-neoplastic hematopoietic stem cells can lead to false-positive ctDNA results, which can be reduced by advanced bioinformatics analysis or comparison of ctDNA sequencing with leukocytes and/or matched tumor tissue (Steensma et al., 2015; Snyder et al., 2016). These mutations represent a confounding factor when analyzing actual tumor variants in the absence of white blood cell (WBC) control samples (Razavi et al., 2019). Therefore, additional NGS analysis of leukocytes is recommended to rule out CHIP-related variants, especially in the case of MRD or early cancer detection.

Agreement between ctDNA and tissue-based NGS results is typically defined as the presence or absence of identical genomic alterations in a single gene on both molecular platforms. The main reasons for inconsistent blood and tissue detection are biopsy location and time, different DNA shedding, tumor heterogeneity, and epigenetic modifications. Lack of standardization between ctDNA tests is another barrier, which limits the understanding of the available results. Inconsistent ctDNA results may be the result of several variables, including the time of sample collection, sample collection process, sample storage method, library construction process, utilization of unique molecular identifiers and bioinformatic analysis.

Accurate risk assessment and adjuvant therapy are very important for cancer patients. ctDNA testing can accurately identify the MRD after primary tumor resection, and thus identify the patient population that needs further adjuvant chemotherapy, so as to avoid unnecessary additional treatment. In addition, determining the duration of adjuvant therapy based on ctDNA clearance can help reduce adverse reactions. However, many researchers also suggested that adjuvant therapy based on negative ctDNA testing should not be excluded due to the low standardization of ctDNA detection procedures and the limitations of ctDNA testing techniques.

Although preliminary data on the clinical application of ctDNA in MRD detection is promising, most of the studies that provide evidence to support it are small, limited in scope and require validation using large independent cohorts (Corcoran and Chabner, 2018; Heitzer et al., 2019). It is only through these further studies that we can solve the next important question of whether acting on positive ctDNA MRD results can improve clinical outcomes or whether ctDNA MRD can be used to more precisely guide adjuvant therapy.

## 5 Conclusion

Overall, MRD aids in the management of cancer at all stages, including screening, guiding adjuvant treatment, predicting relapse early, initiating systemic treatment and monitoring response, and genotyping resistance. Liquid biopsy, espesially ctDNA, can be used as an alternative to tumor tissue detection, especially when tissue biopsy is not feasible or time does not permit. New technologies are being developed, such as methylation pattern-based sequencing which have the potential to optimize ctDNA detection for use in a wide range of scenarios. In the future, we need to carry out more intervention studies to provide stronger evidence support for the application of MRD detection methods, so as to achieve the purpose of integration with clinical routine. Through the monitoring of ctDNA, the therapeutic regimen can be adjusted in time, and the treatment effect can be improved to maximize the survival time of patients.
